# Reduced protein kinase C delta in a high molecular weight complex in mitochondria and elevated creatine uptake into Barth syndrome B lymphoblasts

**DOI:** 10.20517/jtgg.2024.11

**Published:** 2024-05-29

**Authors:** Edgard M. Mejia, Genevieve C. Sparagna, Donald W. Miller, Grant M. Hatch

**Affiliations:** 1 Department of Pharmacology and Therapeutics, University of Manitoba, Winnipeg, MB R3E 0T6, Canada.; 2 Department of Medicine, Division of Cardiology, University of Colorado Anschutz Medical Center, Aurora, CO 80045, USA.; 3 Children’s Hospital Research Institute of Manitoba, Winnipeg, MB R3E 3P4, Canada.

**Keywords:** Barth syndrome, TAFFAZIN, protein kinase C delta, B lymphoblasts, mitochondria, cardiolipin, creatine uptake, monolysocardiolipin

## Abstract

**Aim:**

Barth syndrome (BTHS) is a rare X-linked genetic disease in which mitochondrial oxidative phosphorylation is impaired due to a mutation in the *TAFAZZIN* gene. The protein kinase C delta (PKCδ) signalosome exists as a high molecular weight complex in mitochondria and controls mitochondrial oxidative phosphorylation.

**Method:**

Here, we examined PKCδ levels in mitochondria of aged-matched control and BTHS patient B lymphoblasts and its association with a higher molecular weight complex in mitochondria.

**Result:**

Immunoblot analysis of blue-native polyacrylamide gel electrophoresis mitochondrial fractions revealed an increase in total PKCδ protein expression in BTHS lymphoblasts compared to controls. In contrast, PKCδ associated with a higher molecular weight complex was markedly reduced in BTHS patient B lymphoblasts compared to controls. Given the decrease in PKCδ associated with a higher molecular weight complex in mitochondria, we examined the uptake of creatine, a compound whose utilization is enhanced upon high energy demand. Creatine uptake was markedly elevated in BTHS lymphoblasts compared to controls.

**Conclusion:**

We hypothesize that reduced PKCδ within this higher molecular weight complex in mitochondria may contribute to the bioenergetic defects observed in BTHS lymphoblasts and that enhanced creatine uptake may serve as one of several compensatory mechanisms for the defective mitochondrial oxidative phosphorylation observed in these cells.

## INTRODUCTION

Barth syndrome (BTHS) is a rare X-linked genetic disease caused by a mutation in the *TAFAZZIN* gene localized on chromosome Xq28.1^2[1–3]^. BTHS is characterized by cardiomyopathy, skeletal myopathy, growth retardation, neutropenia, and frequently 3-methylglutaconic aciduria. At the cellular level, BTHS patients exhibit impaired mitochondrial oxidative phosphorylation. The *TAFAZZIN* gene product is a transacylase enzyme involved in the remodeling of the mitochondrial phospholipid cardiolipin (CL) from monolysocardiolipin (MLCL)^[4,5]^. Hence, mutations in *TAFAZZIN* result in reduced CL, elevated MLCL, and impairment in oxidative phosphorylation^[1–3,6]^. In several studies, Epstein-Barr virus transformed lymphoblasts from patients have been used to examine BTHS metabolic pathology^[6–9]^.

Protein kinase C delta (PKCδ) is a signaling kinase that regulates many cellular responses and is controlled via multi-site phosphorylation^[10–13]^. The PKCδ pathway adjusts the fuel flux from glycolytic sources to the intensity of mitochondrial respiration, thus controlling mitochondrial oxidative phosphorylation. In mitochondria, the PKCδ signalosome exists in a high molecular weight complex, which includes cytochrome *c* as the upstream driver of PKCδ, the adapter protein p66Shc as the assembly platform, and retinol^[12,14]^. All four components are required for activation of PKCδ signaling in mitochondria. We previously demonstrated that PKCδ phosphorylation was altered on several sites in BTHS patient B lymphoblasts compared to control patient B lymphoblasts^[15]^. Given that PKCδ is involved in B lymphocyte differentiation and cell fate^[16]^ and that altered phosphorylation of PKCδ may impact its activation, it is possible that PKCδ associated with a higher molecular weight complex is altered in mitochondria of BTHS B lymphoblasts.

Creatine is an amino acid derivative that, upon entrance into cells, is phosphorylated to phosphocreatine and used as an energy buffer. For example, during increased energy demand, ATP is rapidly resynthesized from ADP and phosphocreatine. Thus, creatine uptake is required to support phosphocreatine generation. Since oxidative phosphorylation is impaired in BTHS B lymphoblasts, it is possible that enhanced creatine uptake may occur as a compensatory mechanism to maintain energy metabolism, as observed with other metabolites such as glucose^[17]^.

In this study, we demonstrate for the first time that PKCδ is associated with a higher molecular weight complex in B lymphoblast mitochondria but that its association with this higher molecular weight complex is reduced in BTHS patient B lymphoblasts mitochondria compared to age-matched controls in spite of an increase in overall PKCδ protein expression. We hypothesize that the lack of PKCδ within this high molecular weight complex may contribute to defective mitochondrial PKCδ signaling and thus to the bioenergetic defects observed in BTHS cells. Moreover, we observe enhanced creatine uptake into BTHS patient B lymphoblasts compared to control cells. We hypothesize that enhanced creatine uptake may, in part, contribute as a compensatory mechanism to maintain energy metabolism in BTHS B lymphoblasts.

## MATERIALS AND METHODS

Epstein-Barr virus transformed BTHS B lymphoblasts from 4- to 9-year-old males and male age-matched control lymphoblasts were graciously provided by Dr. Richard Kelley, Kennedy Kreiger Institute, Baltimore, MD., and obtained from Coreill Institute (Camden, NJ) and were cultured as previously described^[9]^. [^14^C]Creatine was obtained from American Radiochemicals Inc. (Burnaby, BC, Canada, Catalog number ARC 0176-50 μCi). Ecolite scintillant was obtained from ICN Biochemicals (Montreal, Quebec, Canada). All other chemicals were of American Chemical Society (ASC) grade and obtained from either Sigma Aldrich Canada Ltd. (Oakville, ON, Canada) or Thermo Fisher Scientific (Carlsbad, CA, USA).

Electrospray ionization mass spectrometry (ESI-MS) coupled with high-performance liquid chromatography (HPLC) mass spectrometry of cardiolipin (CL) and monolysocardiolipin (MLCL) from cell lysates was performed as described^[18]^. Mitochondrial fractions were isolated using the mitochondrial isolation kit from Abcam (Toronto, ON, Canada, Catalog number ab110170). Mitochondrial protein content was determined using the M protein assay kit (Mississauga, ON, Canada). For Blue-Native polyacrylamide gel electrophoresis (BN-PAGE) analysis, mitochondrial protein (80 μg) was treated with 0.2% digitonin and then separated on a 3%−12% gradient gel as described^[9]^. Immunoblot analysis of the gel was performed using anti-PKCδ antibody (1:1,000) (Abcam, Toronto, ON, Canada) as described^[19]^. PKCδ was visualized using the Amersham Enhanced Chemiluminescence Western blotting detection system (VWR, Mississauga, ON, Canada). Band intensity was quantified using Image J software. Citrate synthase activity was measured using the citrate synthase assay kit (Sigma-Aldrich, Oakville, ON, Canada, Catalog number CS0720).

Cells were cultured in RPMI 1,640 medium containing 10% fetal bovine serum and 1% antimycotic and antibiotic solution and incubated at 37 °C in 5% CO_2_ until used. Cells were incubated with 2 mL medium containing 0.1 μM [^14^C]Creatine (specific activity 50–60 mCi/mmol) for up to 60 min. At the indicated time points, the medium was removed and cells washed twice with 2 mL of ice-cold PBS. The PBS was removed and 2 mL of methanol:water (1:1 v/v) was added. The cells were harvested using a rubber policeman and put into test tubes. The mixture was vortexed, and a 50 μL aliquot was taken for protein determination and a 50 μL aliquot taken for determination of radioactivity. Data are expressed as mean ± standard deviation of the mean. Comparisons between control and BTHS patient lymphoblasts were performed by unpaired, two-tailed Student’s *t*-test. A probability value of *P* < 0.05 was considered significant.

## RESULTS

**Figure 1.**
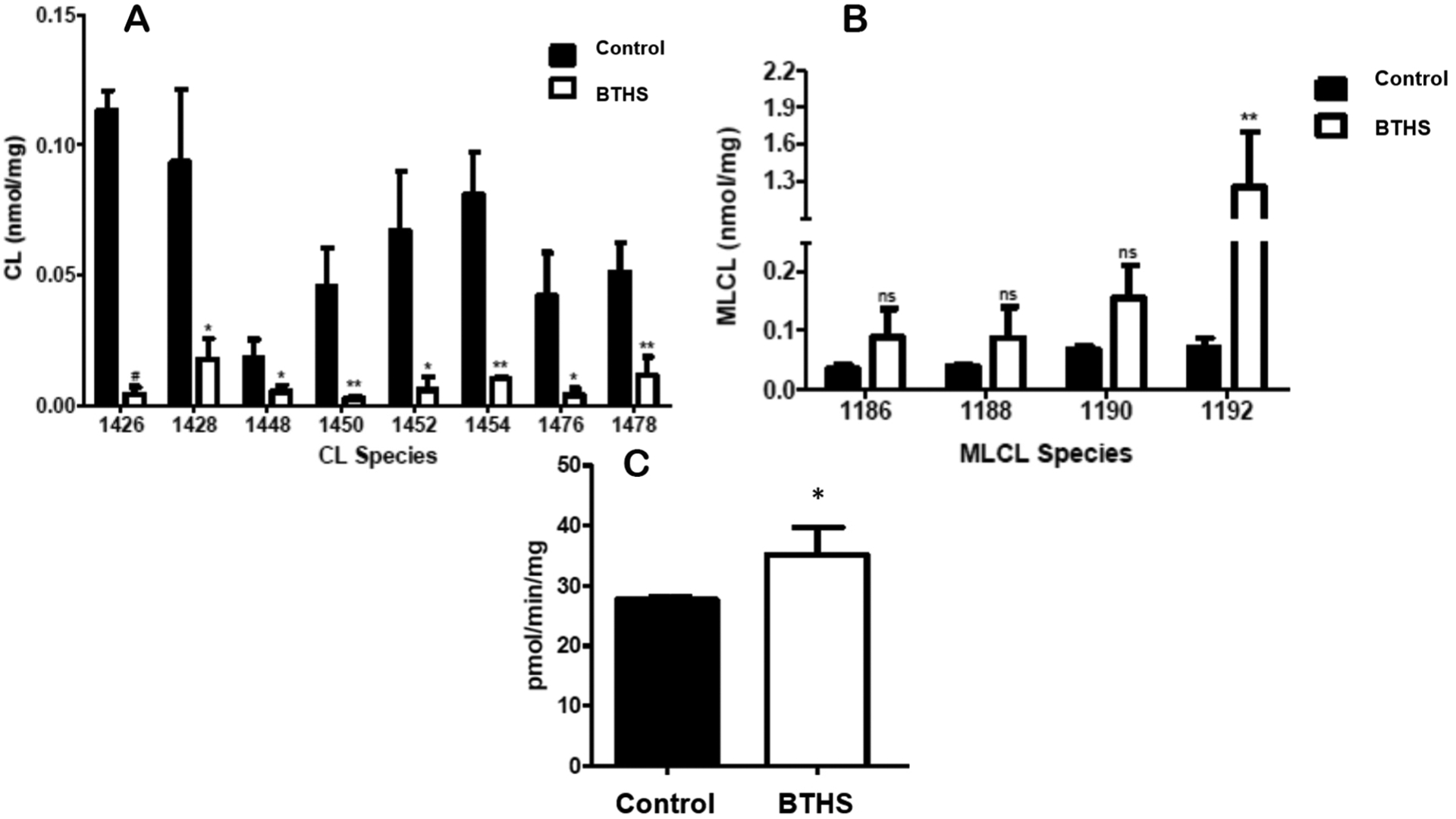
CL levels are reduced and trioleoyl-MLCL and citrate synthase activity elevated in BTHS lymphoblasts. Quantification of the major CL. (A) and Major MLCL (B) Fatty acyl molecular species in age-matched control and BTHS lymphoblasts as described in Materials and Methods. (C) Mitochondrial fractions were prepared from age-matched control and BTHS lymphoblasts and citrate synthase activity determined as described in Materials and Methods. Data represent the mean + SD, *n* = 4. ^#^*P* < 0.001; **P < 0.01; *P < 0.05; ns: not significant.

All major molecular species of CL were significantly reduced in BTHS lymphoblasts compared to age-matched control lymphoblasts [Figure 1A]. This reduction in CL molecular species was accompanied by a general, but not significant, increase in most major MLCL species. In contrast, a > 20-fold increase (*P* < 0.01) in trioleoyl-MLCL [mass/charge (m/z) 1192] molecular species was observed in BTHS lymphoblasts compared to age-matched control lymphoblasts [Figure 1B].

BTHS patient lymphoblasts exhibited abnormally increased mitochondrial mass^[7,8]^. To confirm this, mitochondrial fractions were prepared and citrate synthase activity determined. Citrate synthase activity was elevated 20% (*P* < 0.05) in BTHS lymphoblasts compared to age-matched control cells [Figure 1C]. Thus, the reduction in CL, increase in MLCL and MLCL/CL ratio, and increase in citrate synthase activity were consistent with that observed in BTHS patient B lymphoblast cells.

**Figure 2.**
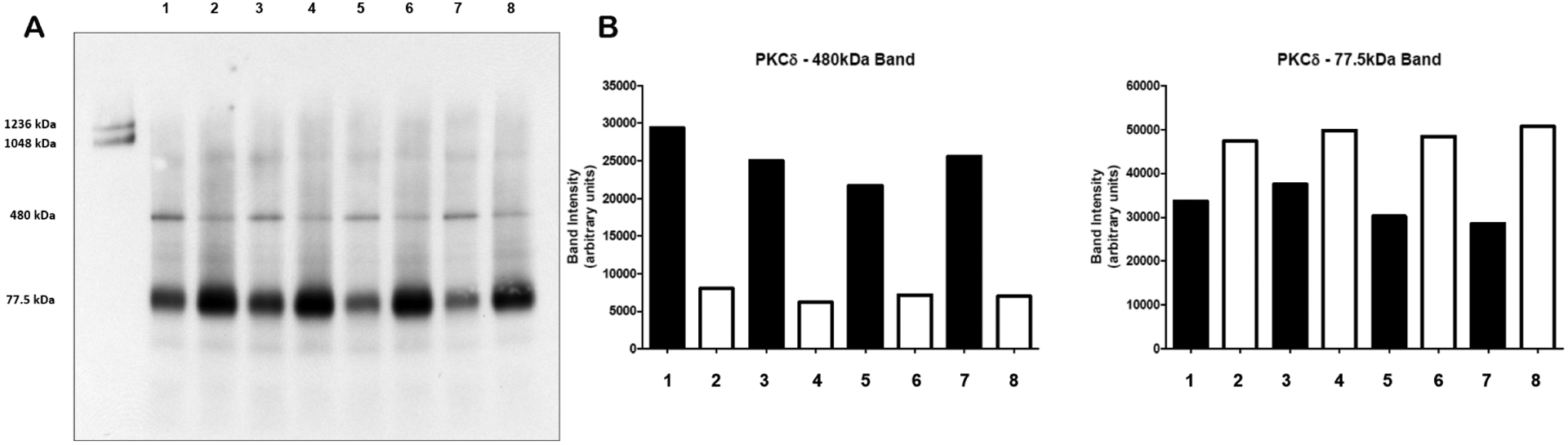
BTHS lymphoblasts exhibit reduced PKCδ associated with a higher molecular weight complex in mitochondria. Mitochondrial fractions were prepared from age-matched control and BTHS lymphoblasts and subjected to BN-PAGE followed by immunoblot analysis of PKCδ. (A) Age-matched control (lanes 1, 3, 5 and 7); BTHS lymphoblasts (lanes 2, 4, 6 and 8). Molecular mass markers are in the first lane and indicated on the left. (B) Densitometry quantification of PKCδ.

We previously observed that PKCδ phosphorylation was altered on several sites examined in BTHS lymphoblasts^[15]^. Since altered phosphorylation may affect PKCδ activation^[13]^, we examined if this was related to altered PKCδ associated with higher molecular weight complex in mitochondria of BTHS patient lymphoblasts. Mitochondrial fractions were subjected to BN-PAGE followed by immunoblot analysis for determination of PKCδ levels. The two upper bands indicated on the left of the blot are molecular mass markers at 1,236 and 1,048 kDa, respectively [Figure 2A]. The level of PKCδ located on the gel at a predicted molecular mass near 77.5 kDa was elevated by 1.5-fold (*P* < 0.05) in BTHS lymphoblasts compared to age-matched control cells. In contrast, the level of PKCδ located on the gel at approximately 480 kDa was reduced by 72% (*P* < 0.01) in BTHS lymphoblasts compared to age-matched control cells [Figure 2B]. Thus, BTHS lymphoblasts exhibit elevated expression of PKCδ but reduced PKCδ associated with a higher molecular weight complex in mitochondria.

**Figure 3.**
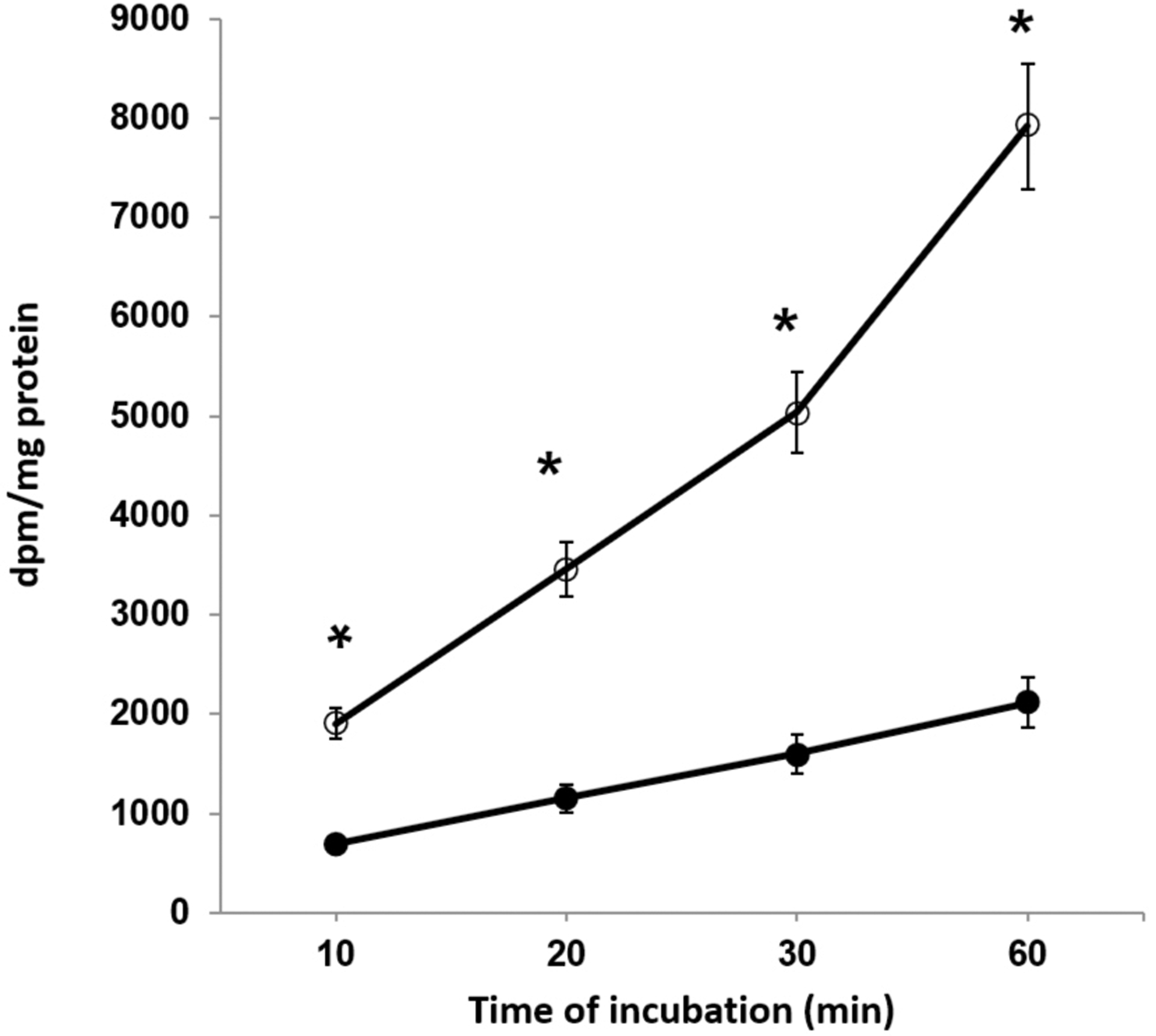
BTHS lymphoblasts exhibit elevated creatine uptake. Control (closed symbols) and BTHS (open symbols) B lymphoblasts were incubated with [^14^C]Creatine for up to 60 min and radioactivity incorporated into cells determined. Data represent the mean + SD, *n* = 4. *P < 0.001.

Since oxidative phosphorylation is impaired in BTHS B lymphoblasts^[9]^, it is possible that enhanced creatine uptake may occur as a compensatory mechanism to maintain energy metabolism as observed with other metabolites such as glucose^[17]^. Control and BTHS lymphoblasts were incubated with [^14^C]Creatine for up to 60 min and radioactivity incorporated into cells determined. [^14^C]Creatine incorporation into BTHS lymphoblasts was markedly elevated compared to control cells [Figure 3]. Thus, BTHS lymphoblasts exhibit enhanced creatine uptake.

## DISCUSSION

BTHS is a rare X-linked genetic disease and is the only known disease in which the specific biochemical defect is a reduction in CL and accumulation of MLCL^[1–3]^. We observed a reduction in all major molecular species of CL in BTHS lymphoblasts accompanied by a > 20-fold elevation in trioleoyl-MLCL. Previous studies demonstrated an increase in abnormal mitochondrial mass in BTHS patient lymphoblasts^[7,8]^. We confirmed this observation in our BTHS patient lymphoblasts through an increase in mitochondrial citrate synthase activity.

BTHS lymphoblasts exhibit impaired oxidative phosphorylation, elevated oxidative stress, and increased reactive oxygen species^[8,9]^. It was recently demonstrated that accumulation of MLCL in several BTHS models forms a peroxidase complex with cytochrome c capable of oxidizing polyunsaturated fatty acid-containing lipids^[20]^. The authors of that study showed that accumulation of MLCL facilitates the formation of anomalous MLCL-cytochrome c peroxidase complexes and hypothesized that peroxidation of polyunsaturated fatty acid phospholipids is the primary pathogenic mechanism of BTHS. Indeed, oxidative stress is known to induce the expression of PKCδ^[21]^. We observed increased protein expression of 77.5 kDa PKCδ in the mitochondria of BTHS lymphoblasts compared to controls. The elevated PKCδ levels observed might serve as a compensatory mechanism to increase ATP production in BTHS cells through PKCδ signaling^[12]^. Additionally, elevated expression of PKCδ promotes mitochondrial proliferation^[21]^. As indicated above, abnormal proliferation of BTHS lymphoblast mitochondria has been observed previously^[7,8]^. Phosphorylation of PKCδ is required for its activation^[13]^. We previously observed an alteration in the phosphorylation of PKCδ in BTHS lymphoblasts^[15]^. Hence, altered phosphorylation of PKCδ might contribute to an attenuated mitochondrial PKCδ signaling in these cells.

The molecular components that mediate PKCδ signaling in mitochondria are beginning to emerge. Mitochondria contain a high molecular weight functional complex, which includes cytochrome *c* as the upstream driver of PKCδ, and it uses the adapter protein p66Shc as the assembly platform with vitamin A (retinol)^[12,14,22]^. All four partners are required for functional PKCδ signaling. BN-PAGE immunoblot analysis of mitochondrial proteins not only has the advantage of probing for expression of individual proteins but may additionally be used to detect if these proteins are associated with higher molecular weight complexes. Using this approach, we observed a reduction in PKCδ associated with a higher molecular weight complex in BTHS B lymphoblasts mitochondria. It is possible that the decreased association of PKCδ with the high molecular weight complex was associated with accumulation of MLCL in our BTHS lymphoblasts and that the increased ratio of MLCL to CL may affect inner membrane structural integrity such that the high molecular weight complex dissociates. Previous studies have demonstrated that MLCL-protein interactions compromise the stability of the protein-dense mitochondrial inner membrane^[23]^.

The PKCδ/retinol complex signals the pyruvate dehydrogenase complex for enhanced flux of pyruvate into the Krebs cycle^[12,14]^. Interestingly, in the UK BTHS NHS clinic, almost half of the BTHS boys examined showed signs of Vitamin A deficiency (Nicol Clayton: https://www.youtube.com/watch?v=wNDr_oCTJ7A). However, supplementation with Vitamin A did not increase plasma levels. This was not because tissue levels were low but possibly due to increased levels of Vitamin A (retinyl esters) in chylomicrons. It is possible that this unique observation is coupled to defective mitochondrial PKCδ signaling, which might contribute to reduced ATP production in the Krebs cycle through alteration in the mitochondrial PKCδ/retinol signaling complex and contribute to the multitude of bioenergetic defects observed in BTHS. However, it is unknown whether the decreased association of PKCδ within the high molecular weight complex in Barth Syndrome is a cause of mitochondrial dysfunction or an effect of mitochondrial dysfunction.

Creatine is an important energy metabolite that is used as an energy buffer. Impaired mitochondrial oxidative phosphorylation, as seen in BTHS, may require increased energy demand from alternative sources such as ATP synthesis from enhanced glucose uptake and oxidation or ATP resynthesis from ADP and phosphocreatine. Interestingly, creatine supplementation has been shown to increase glucose uptake and oxidation and adenosine monophosphate kinase (AMPK) phosphorylation in skeletal muscle cells^[24]^. We previously reported that increased AMPK phosphorylation and its activation accompanied elevated glucose uptake in BTHS B lymphoblasts^[17]^. Enhanced glucose uptake in TAFZZIN-deficient cells may additionally be linked to the upregulation of pyruvate dehydrogenase 4 mediated through AMPK activation and transcriptional upregulation by forkhead box protein O1^[25]^. Moreover, creatine kinase has been shown to be mildly elevated in the plasma of some BTHS patients^[26]^. Thus, enhanced creatine uptake might be required to support phosphocreatine generation if creatine kinase was depleted in cells of these patients. In the current study, creatine uptake was significantly enhanced in BTHS B lymphoblasts compared to controls. Interestingly, the human creatine transporter (CRTR) gene was shown to be localized on Xq28 and, at one time, was hypothesized to be a candidate gene for BTHS and infantile cardiomyopathy^[27]^. However, a subsequent study by Sylvia Bione identified the actual locus of the human *TAFAZZIN* gene to be Xq28.12^[28]^. Whether creatine supplementation improves the health of BTHS patients is unknown. A previous study indicated that creatine supplementation in humans improved performance during exercise of high to maximal intensity^[29]^.

It would be intriguing to examine creatine uptake and whether localization of PKCδ within a higher molecular weight complex in mitochondria is impaired in cells of multi-system mitochondrial disease patients with CL synthase (CRLS1) dysfunction in which loss of CL and phosphatidylglycerol accumulation results in fragmented mitochondrial morphology and bioenergetic dysfunction^[30]^. This might address whether the accumulation of MLCL is responsible for our observations.

Although much has been learned on regulation of cellular metabolism and the immune response from Epstein-Barr virus transformed B lymphoblasts since the discovery of the first human tumor virus by Epstein, Achong and Barr 60 years ago^[31]^, caution should be exercised in interpretation of our results as the transformed nature of these cells has been shown to modify both expression and alternative splicing of host cell genes^[32,33]^.

## CONCLUSION

We conclude that impaired localization of PKCδ within a higher molecular weight complex in mitochondria may contribute to the bioenergetic defects observed in BTHS B lymphoblasts and enhanced creatine uptake may serve as a compensatory mechanism for the defective mitochondrial oxidative phosphorylation observed in these cells.

## Data Availability

All data supporting the findings can be found within the manuscript.

## References

[1] Barth PG, Scholte HR, Berden JA, An X-linked mitochondrial disease affecting cardiac muscle, skeletal muscle and neutrophil leucocytes. J Neurol Sci 1983;62:327–55. 6142097 10.1016/0022-510x(83)90209-5

[2] Kelley RI, Cheatham JP, Clark BJ, X-linked dilated cardiomyopathy with neutropenia, growth retardation, and 3-methylglutaconic aciduria. J Pediatr 1991;119:738–47. 1719174 10.1016/s0022-3476(05)80289-6

[3] Zegallai HM, Hatch GM. Barth syndrome: cardiolipin, cellular pathophysiology, management, and novel therapeutic targets. Mol Cell Biochem 2021;476:1605–29. 33415565 10.1007/s11010-020-04021-0

[4] Xu Y, Malhotra A, Ren M, Schlame M. The enzymatic function of tafazzin. J Biol Chem 2006;281:39217–24. 17082194 10.1074/jbc.M606100200

[5] Xu Y, Kelley RI, Blanck TJ, Schlame M. Remodeling of cardiolipin by phospholipid transacylation. J Biol Chem 2003;278:51380–5. 14551214 10.1074/jbc.M307382200

[6] Valianpour F, Mitsakos V, Schlemmer D, Monolysocardiolipins accumulate in Barth syndrome but do not lead to enhanced apoptosis. J Lipid Res 2005;46:1182–95. 15805542 10.1194/jlr.M500056-JLR200

[7] Xu Y, Sutachan JJ, Plesken H, Kelley RI, Schlame M. Characterization of lymphoblast mitochondria from patients with Barth syndrome. Lab Invest 2005;85:823–30. 15806137 10.1038/labinvest.3700274

[8] Gonzalvez F, D’Aurelio M, Boutant M, Barth syndrome: cellular compensation of mitochondrial dysfunction and apoptosis inhibition due to changes in cardiolipin remodeling linked to tafazzin (TAZ) gene mutation. Biochim Biophys Acta 2013;1832:1194–206. 23523468 10.1016/j.bbadis.2013.03.005

[9] Mejia EM, Zegallai H, Bouchard ED, Banerji V, Ravandi A, Hatch GM. Expression of human monolysocardiolipin acyltransferase-1 improves mitochondrial function in Barth syndrome lymphoblasts. J Biol Chem 2018;293:7564–77. 29563154 10.1074/jbc.RA117.001024 PMC5961033

[10] Reyland ME, Jones DN. Multifunctional roles of PKCδ: opportunities for targeted therapy in human disease. Pharmacol Ther 2016;165:1–13. 27179744 10.1016/j.pharmthera.2016.05.001 PMC5116389

[11] Qvit N, Mochly-Rosen D. The many hats of protein kinase Cδ: one enzyme with many functions. Biochem Soc Trans 2014;42:1529–33. 25399565 10.1042/BST20140189 PMC4383467

[12] Kim YK, Hammerling U. The mitochondrial PKCδ/retinol signal complex exerts real-time control on energy homeostasis. Biochim Biophys Acta Mol Cell Biol Lipids 2020;1865:158614. 31927141 10.1016/j.bbalip.2020.158614 PMC7347429

[13] Yang Q, Langston JC, Tang Y, Kiani MF, Kilpatrick LE. The role of tyrosine phosphorylation of protein kinase C delta in infection and inflammation. Int J Mol Sci 2019;20:1498. 30917487 10.3390/ijms20061498 PMC6471617

[14] Acin-Perez R, Hoyos B, Zhao F, Control of oxidative phosphorylation by vitamin A illuminates a fundamental role in mitochondrial energy homoeostasis. FASEB J 2010;24:627–36. 19812372 10.1096/fj.09-142281 PMC2812036

[15] Agarwal P, Cole LK, Chandrakumar A, Phosphokinome analysis of Barth syndrome lymphoblasts identify novel targets in the pathophysiology of the disease. Int J Mol Sci 2018;19:2026. 30002286 10.3390/ijms19072026 PMC6073761

[16] Mecklenbräuker I, Saijo K, Zheng NY, Leitges M, Tarakhovsky A. Protein kinase Cdelta controls self-antigen-induced B-cell tolerance. Nature 2002;416:860–5. 11976686 10.1038/416860a

[17] Mejia EM, Zinko JC, Hauff KD, Xu FY, Ravandi A, Hatch GM. Glucose uptake and triacylglycerol synthesis are increased in barth syndrome lymphoblasts. Lipids 2017;52:161–5. 28097490 10.1007/s11745-017-4232-7

[18] Sparagna GC, Johnson CA, McCune SA, Moore RL, Murphy RC. Quantitation of cardiolipin molecular species in spontaneously hypertensive heart failure rats using electrospray ionization mass spectrometry. J Lipid Res 2005;46:1196–204. 15772420 10.1194/jlr.M500031-JLR200

[19] Chang W, Zhang M, Chen L, Hatch GM. Berberine inhibits oxygen consumption rate independent of alteration in cardiolipin levels in H9c2 cells. Lipids 2017;52:961–7. 28942573 10.1007/s11745-017-4300-z

[20] Kagan VE, Tyurina YY, Mikulska-Ruminska K, Anomalous peroxidase activity of cytochrome c is the primary pathogenic target in Barth syndrome. Nat Metab 2023;5:2184–205. 37996701 10.1038/s42255-023-00926-4 PMC11213643

[21] Lee CF, Chen YC, Liu CY, Wei YH. Involvement of protein kinase C delta in the alteration of mitochondrial mass in human cells under oxidative stress. Free Radic Biol Med 2006;40:2136–46. 16785027 10.1016/j.freeradbiomed.2006.02.008

[22] Acin-Perez R, Hoyos B, Gong J, Regulation of intermediary metabolism by the PKCδ signalosome in mitochondria. FASEB J 2010;24:5033–42. 20798245 10.1096/fj.10-166934 PMC2992363

[23] Duncan AL. Monolysocardiolipin (MLCL) interactions with mitochondrial membrane proteins. Biochem Soc Trans 2020;48:993–1004. 32453413 10.1042/BST20190932 PMC7329354

[24] Ceddia RB, Sweeney G. Creatine supplementation increases glucose oxidation and AMPK phosphorylation and reduces lactate production in L6 rat skeletal muscle cells. J Physiol 2004;555:409–21. 14724211 10.1113/jphysiol.2003.056291 PMC1664837

[25] Liang Z, Ralph-Epps T, Schmidtke MW, Upregulation of the AMPK-FOXO1-PDK4 pathway is a primary mechanism of pyruvate dehydrogenase activity reduction and leads to increased glucose uptake in tafazzin-deficient cells. Sci Rep 2024;14:11497. 38769106 10.1038/s41598-024-62262-1 PMC11106297

[26] Spencer CT, Bryant RM, Day J, Cardiac and clinical phenotype in Barth syndrome. Pediatrics 2006;118:e337–46. 16847078 10.1542/peds.2005-2667

[27] Sandoval N, Bauer D, Brenner V, The genomic organization of a human creatine transporter (CRTR) gene located in Xq28. Genomics 1996;35:383–5. 8661155 10.1006/geno.1996.0373

[28] Bione S, D’Adamo P, Maestrini E, Gedeon AK, Bolhuis PA, Toniolo D. A novel X-linked gene, G4.5. is responsible for Barth syndrome. Nat Genet 1996;12:385–9. 8630491 10.1038/ng0496-385

[29] Casey A, Greenhaff PL. Does dietary creatine supplementation play a role in skeletal muscle metabolism and performance? Am J Clin Nutr 2000;72:607S–17S. 10919967 10.1093/ajcn/72.2.607S

[30] Lee RG, Balasubramaniam S, Stentenbach M, Deleterious variants in CRLS1 lead to cardiolipin deficiency and cause an autosomal recessive multi-system mitochondrial disease. Hum Mol Genet 2022;31:3597–612. 35147173 10.1093/hmg/ddac040 PMC9616573

[31] Young LS. Epstein-Barr virus at 60. Nature 2024;627:492–4. 38480942 10.1038/d41586-024-00653-0

[32] Manet E, Polvèche H, Mure F, Modulation of alternative splicing during early infection of human primary B lymphocytes with epstein-barr virus (EBV): a novel function for the viral EBNA-LP protein. Nucleic Acids Res 2021;49:10657–76. 34530456 10.1093/nar/gkab787 PMC8501971

[33] Tang Y, Zhong Y, Fu T, Bioinformatic analysis of differentially expressed genes and identification of key genes in EBV-transformed lymphoblasts. Biomed Pharmacother 2019;116:108984. 31129512 10.1016/j.biopha.2019.108984

